# Medication challenges for patients with severe mental illness: experience and views of patients, caregivers and mental health care workers in Dar es Salaam, Tanzania

**DOI:** 10.1186/s13033-017-0126-6

**Published:** 2017-02-06

**Authors:** Masunga K. Iseselo, Joel Seme Ambikile

**Affiliations:** Department of Clinical Nursing, Muhimbili University of Health and Allied Sciences, P.O.Box 65004, Dar es Salaam, Tanzania

**Keywords:** Psychotropic medication, Mental disorders, Patients, Caregiver

## Abstract

**Background:**

Management of patients with mental disorders is inadequate in the majority of low and middle income countries. The main treatment modality for patients with severe mental disorders in these countries is mainly pharmacological approach. Patients face many challenges in meeting medication needs. In this context, high percentages of individuals who have severe mental disorders are not treated. Regular and adequate supplies of appropriate, safe and affordable medications are some of the important aspects required for provision of quality mental health services. Psychotropic medications are an important component of holistic care that provides treatment options for those suffering from mental illnesses. In Tanzania, mental health services face many challenges including inadequate mental health care providers, infrastructure, and medication supply. Relapse is a common problem among patients attending mental health facilities. This study is aimed at exploring views and experiences of patients, caregivers and mental health care providers on the psychotropic medication in Dar es Salaam, Tanzania

**Methods:**

A qualitative study was conducted, involving two focus group discussions with seven and nine caregivers in each group. Eleven in-depth interviews with four patients and seven mental health care providers at Temeke Municipality, Dar es Salaam, were conducted. Convenient sampling procedure was used to select participants for the study. Discussion and interview guides were used during data collection. Interviews were audio-recorded in Kiswahili with all study participants. The recorded interviews were transcribed and qualitative content thematic analysis was used to analyze data after translation.

**Results:**

Four themes were identified. These include attitudes of patients towards psychotropic medication, availability of psychotropic medications, financial concerns towards psychotropic medications, and coverage of free treatment policy.

**Conclusion:**

The availability and affordability of psychotropic medications to patients are big problems. This was partly attributed to insufficient funds to support the budget of health facilities and technical challenges contributed by both the health facilities and other stakeholders. To improve mental health services in the country, it is important to ensure adequate supply of psychotropic medications in the health facilities. Access to psychotropic medications is essential in addressing the public health problem of untreated mental illnesses. These findings call for the government and other stakeholders to increase funding for essential psychotropic medications.

## Background

Treatment of patients with severe mental illness is inadequate in most low and middle income countries (LMICs). High percentages of individuals who have severe mental disorders such as bipolar disorder, major depressive disorder and schizophrenia, remain untreated [1]. In addition to this disease burden, LMICs face many challenges in meeting mental health needs in their regions. Emphasis has been put on the role for both psychosocial and pharmacological management of mental illnesses [2]. However, psychosocial management is almost non-existent and pharmacological therapy is the most common treatment method in many government-funded health facilities [3, 4]. It is also important to note that psychosocial, together with biological treatments, have proven to be effective in low resource settings [5].

One of the biggest challenges to provision of services to the people with mental illness in low and middle income countries is that of ensuring regular and adequate supply of appropriate, safe, and affordable medications. Psychotropic medications are important component of holistic care that provides treatment options for those suffering from mental illnesses [3]. Studies have reported that lack of up-to-date data on the availability of effective and efficacious medications for mental healthcare in these countries is a major problem that inhibits quality patient care [4, 5].

In Tanzania, most health facilities are not equipped with enough resources, which include a continuous supply of essential psychotropic medications that could help improve quality of mental health care.

Mental health services are provided free of charge. However, relapse problems with mental health clients attending the district health facilities are very common. The most important factors contributing to relapse include non-adherence to medication, side effects, and inadequate knowledge on management of symptoms among patients and family members [6–8].

The free services for mental health is not realised in most health services in low and middle income countries, especially when it comes to availability of cost effective medications. Studies in other countries report that direct out-of-pocket payment by patients is required in order to get prescribed medication which is not available for free in the government pharmacies [4, 5, 9]. The need of medication is important to control symptoms, which may be frustrating to the patient and caregiver if not controlled. A study in Tanzania revealed difficulties in accessing medication by the patient due to high cost of getting them in pharmacies outside the hospital [10].

For the benefit of the patient with mental disorders, health systems have an important role to play in facilitating access to psychotropic medicine in any country. A cross sectional analysis of 63 low and middle income countries suggested that strengthening particular aspects of mental health systems may lead to improved availability of psychotropic medicines, and that the overall country development is associated with medication affordability [11]. This implies that governments have not invested much in mental health systems. Evidence reveals that there is minimal government spending on mental health services in most LMICs (1–2% of the health budget), despite having large numbers of people in need of the services [12].

The existence of a comprehensive national mental health plan in most of the countries has been found to facilitate improvement in access to psychotropic medications [13]. This plan guides countries on how the health system can be more user friendly, especially in accessing mental health services. Additionally, the World Health Organization (WHO) has listed factors that are important for improving access to psychotropic medications. These include rational selection of the medicine, affordable price, longstanding financing, and reliable health and supply systems [14]. In most of the countries, supply of psychotropic medication to rural health facilities is not sufficient. There are many factors that contribute to inadequate supplies of psychotropic medicine in most of government health facilities. Inadequate funding, lack of priorities, perception and past experience of prescribers has been reported to be important factors in many countries in LMIC [9].

This study explored the experiences and views of patients, caregivers and health care providers in meeting medication needs of people with mental illness.

## Methods

### Study design

This was a qualitative study that explored experiences and views of patients, health care providers and caregivers on medication needs of patients with mental disorders.

### Study site

The study was conducted in Temeke Municipal Hospital which is one of the hospitals in Temeke District, Dar es Salaam. Dar es Salaam is very fast growing and is the largest commercial city in Tanzania with the population of more than six million. Temeke district had a population of 1,368,881 as per demographic and population Census of 2012. Socio-economic characteristics of this area that are likely to influence the occurrence of mental health problems/disorders includes the fact that most of youth from other regions in the country move to this area with high expectation of getting employment. However, most of them are unable to obtain employment and as a result of this disappointment become involved with illegal drug business and drug abuse.

### Sample size and sampling procedure

A convenient sampling was used to obtain four patients, sixteen caregivers, and seven health care providers. Patients were recruited through Temeke Municipal Hospital as they attended the mental health clinic. Caregivers were obtained through patients as they escorted them to the clinic. The mental health coordinators were obtained in their respective offices. The sample size was determined by saturation point, i.e. when there was no new information obtained from the informants.

The following inclusion criterion was used to get participants:

1. Health care providers who have been working in the mental health clinics for at least 3 years.
2. Patients who had a chronic mental illness and who were mentally stable at the time of interview (i.e. with insight and able to communicate properly). These were identified using a quick mental state evaluation, which is a standard mental health test.
3. Main caregivers who stayed with a patient with mental illness for at least 6 months.
4. Participants were aged 18 years and above.

Exclusion criteria included the following:

1. Caregivers with communication problems.
2. Health care providers who were on leave during data collection.

### Ethical consideration

The ethical clearance of the study was obtained from the Directorate of Research and Publication Committee of the Muhimbili University of Health and Allied Sciences. Permission to conduct the study was sought from the Regional Administrative Officer and lower authorities in the region including district medical officer and hospital in-charge. Participants were informed about the study procedures. Their role and expectations in the study as participants were clearly stated. Written informed consent was sought from participants. The consent was obtained prior to interview sessions. For people with mental illness, informed consent was first sought from their caregivers. All participants agreed to participate and signed the consent form. They also consented to be audio recorded during the discussion and interview.

Their role and expectations in the study as participants were clearly stated. Confidentiality was guaranteed by identifying participants using numbers and not their names. They were also assured that the audiotapes, transcripts, and written notes containing the participants’ information would be kept well in a safe place and only the researchers would have access to information and would be destroyed later after completion of the study.

### Data collection

Data collection took place on hospital premises. Focus group discussions (FGDs) and in-depth interviews were used during data collection. In-depth interviews were conducted with mental health care workers and coordinators, and patients attending mental health clinics at the health facility. FGDs were conducted among caregivers. The venue for both discussion and interviews was carefully chosen to ensure that it is a quiet place with no distractions. Both groups provided information on their experience and views about challenges encountered on meeting medication needs.

Eleven in-depth interviews including five health care providers, two mental health coordinators and four patients, while two FGD with seven caregivers in FGD 1 and 9 and nine caregivers in FGD 2, participated in discussions. The mental health coordinators were interviewed in their respective offices.

The duration of in-depth interviews was between 30 and 45 min, while for FGDs it was between 45 and 60 min. FGDs were conducted in order to get views from caregivers of patients with mental illness on support they provide at home especially meeting medication needs. Female and males caregivers were interviewed separately to facilitate free expression as a principle of a homogenous group. Questions that were asked during data collection were as follows:

*For mental health care providers and coordinators*

1. What is your experience at the health facility in providing mental health services to patients?
2. How is your health facility prepared to meet psychotropic medication needs of patients?
3. What do you think should be done to improve access to psychotropic medication in your facility?

*For patients*

1. What is your experience in getting mental health services in this hospital, especially with regard to medication?
2. Are you satisfied? And why?
3. What do you think should be done to improve mental health services in this hospital, especially medication?

*For caregivers*

1. What do you think are the needs of a mentally ill patient at home?
2. What are your views about how psychotropic medication needs are met by your patient?
3. What support have you been providing to your patient with regard to medication need and what are the challenges faced?

Each question, in all interviews, was followed by several probes to elicit more information on the matter in question.

Both types of data collection were conducted in Swahili, which is a common language to all participants, and all interviews were digitally audio-recorded. Data that was not easily captured by the audio recorder, such as non-verbal cues and environmental events, was wr downitten in the note book. In case of FGDs, the moderator (researcher) was leading the discussion and kept the conversation flowing. A research assistant was helping to record the interviews and take notes.

### Data analysis

**Fig. 1:**
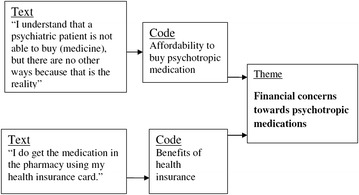
The coding process and formation of themes

All the audio data was transcribed verbatim and then translated into English. The content analysis method, in accordance with qualitative analytical framework, was used to analyse data which involved the researchers reading and re-reading the text. NVivo 10 software was used in organizing data and coding the text. The coded text was filtered and placed in similar contents that formed a tree node. The identified content of the text was entered into memos which were eventually manually organized into codes and themes as in the Fig. 1.

### Trustworthiness

Credibility of the data collected was addressed by employing two methods of data collection i.e. use of FGDs and in-depth interviews, and also, that data was obtained from different sources such as patients, health care workers and caregivers. The interview guide was used to ensure consistency during data collection.

## Results

### Characteristics of participants

The study was comprised of three groups of participants; mental health care workers (MHCW), caregivers (CG) and patients (PT).

The health care workers included two assistant medical officers (AMO), 3 Nurses, one social workers (SW), the district mental health coordinator (DMHC), and the National mental health coordinator (NMHC) (see Table 1). Among the nurses who were interviewed, one was a nursing officer, two were senior nursing officers and one was working as social worker.

**Table 1:** Characteristics of health care workers

Informants	Frequency
Age (years)	
25–34	1
35–44	2
45–54	3
55 and above	1
Sex	
Male	5
Female	2
Cadre	
AMOs	2
Nurses	3
Coordinators	2
Duration of work experience	
3–12 years	2
13–22	3
23–32	2
33 and above	0

Every participant had a working experience of more than 3 years in that position and all were working in the mental health clinic. There were four patients (see Table 2) and sixteen caregivers (see Table 3).

**Table 2:** Patient’s characteristics

Informants	Frequency
Age (years)	
20–29	2
30–39	1
40–49	0
50–59	0
60 and above	1
Sex	
Male	2
Female	2
Marital status	
Married	1
Separated	1
Divorced	0
Single	2
Cohabiting	0
Occupation	
Student	1
Employed	0
Retired	1
Self employed	1
Not employed	1
Diagnosis	
Bipolar disorders	2
Schizophrenia	1
Major depression	1
Years lived with mental illness	
6 months–2 years	1
3–12 years	2
13–22	0
23–32	1
33 and above	0

**Table 3:** Characteristics of caregivers

Informants	Frequency
Age (years)	
20–29	3
30–39	7
40–49	5
50–59	1
60 and above	0
Sex	
Male	7
Female	9
Marital status	
Married	9
Separated	1
Divorced	
Widowed	2
Single	4
Cohabiting	0
Occupation	
Student	0
Employed	2
Retired	0
Self employed	11
Not employed	3
Residence	
Tandika	5
Yombo Buza	3
M/Kijichi	1
Mtongani	2
Mbagala	5
Duration lived with patient	
0–9	11
10–19	4
20–29	1
30 and above	0

### Themes identified

The following themes emerged after data analysis; attitudes of patients towards psychotropic medication, availability of psychotropic medications, *Financial concerns towards psychotropic medications and* coverage of free treatment policy

### Attitudes of patients towards psychotropic medication

Most participants were satisfied with the therapeutic effects of psychotropic medication. Patients and caregivers appreciated the changes experienced or observed just after starting treatment at the health facility. Others, however, had mixed beliefs on the effectiveness of the psychotropic medications. They expected instant therapeutic effect that could bring changes to the patient. When they failed to see these changes immediately, they were likely to seek alternative treatment, especially from traditional healers.

*“Since he began to get medicine this month he has calmed down a bit, as the previous incidences are not available anymore. He is doing fine and has insights”* (FGD 1 CG No 2)

*“……You see, there are those who benefit and those who do not benefit from these medications. Those who feel delayed therapeutic effects they often seek traditional treatment”* (FGD 2 CG No 7)

Caregivers reported being troubled with patients’ symptoms. They were compelled to bring the patient to the hospital when they needed to stabilize patient’s symptoms. They expressed satisfaction with getting psychotropic medication particularly when the symptoms that caused the patients to stay awake during the night disappeared.

*“….then the family members are very careful to ensure that the prescribed medications are taken accordingly. Now the patient is doing fine” (FGD 2 CG No 4)*

The patients also reported that the psychotropic agents prescribed were a good booster of inducing sleep as stated by a patient with bipolar disorder;

*“Before starting to use the medicine I was not able to sleep. I was just staying awake throughout the night but now I am able to sleep”* (Female Pt with BPD)

### Availability psychotropic medications at the health facility

The availability of medications, as expressed by most participants, was a major challenge. Patients, caregivers, health care workers, and coordinators acknowledged that medications were not readily available at the facility due to various reasons including inadequate budget and not being available at the main store in the hospital.

Health care workers working in the hospital expressed the availability of psychotropic medications as unpredictable. Lack of medication sometimes made patients angry and feel that health workers were reluctant to issue medication as described by a nurse:

*“There is no psychotropic medication and sometimes patients become angry and therefore use abusive language towards the health worker, like ‘why don’t you give us medication?’… Sometimes medicines become available but now it has been four or five months with no essential psychotropic medicines. There is no medication for patients with schizophrenia and epilepsy; the medicines that are most often used”* (HCW Nurse)

Another health worker added:

*“We do not get atypical antipsychotics; the antipsychotic that saves here is only CPZ* (Chlopromizine)*. If this medicine is out of store* (not available)*, then you get fever (*confused; not knowing what to do) *because haloperidol itself is very rarely available”* (HCW–AMO)

This problem was noted by the district mental health coordinator, showing the importance of having a continuous supply of medication in the hospital pharmacy due to the fact that most patients do not afford to buy prescribed medications.

*“There is no continuous supply of medicine that patients need to get from the hospital. They are told to go and buy outside the hospital. You find that the patient cannot afford to buy the prescribed medication”* (DMHC).

Caregivers also expressed similar concerns. They explained the challenges they face particularly when they do not get free medications for their patients from the hospital. Atypical antipsychotics, which have fewer side eAttitudes of patients towards psychotropic medication effect profiles, were the most unavailable medications described by the participants. Although these antipsychotics are not frequently prescribed as first line drug, they are rarely available in the government pharmacies. When the medicine is out of stock (not available), patients are instructed to buy from non-governmental pharmacies. One participant whose patient used more than one medication desperately stated;

*“The main challenge here in this hospital is availability of medicine. They just tell you that the medicine is finished, you need to buy and this is not only once, very often. You have to incur the cost which is so high. For example, what I know, my patient uses two types of medicines, I do not know its name, this one is available but the other one, Olanzapine*(drug for treatment of schizophrenia) *is quite unavailable here in the hospital”* (FGD 1 CG No 3).

On the other hand, patients were the most vulnerable population and were directly affected by the shortage of medicine. Patients, especially those who came alone for medicine refill, suffered the most because, with all the risks of transport problems and other urban challenges, they were told to go and look for medication outside the hospitals as expressed by a patient with bipolar disorder:

*“So the chief challenge here is medicine, most of the time we are told to buy medicine because here in the hospital it is not available. Remember, I have come alone but I have to go and look for non*-*government pharmacy to buy my the medication”* (Male Pt with BPD)

#### Reasons for inadequate medication

The procurement process by the hospital and then the ordering by the mental health department was a long process that which was said to be the reason for inadequate availability of medications. It was overtaken by the rapid requirements of the medicine by patients. So the mental health department could order the medicines, but due to the complicated process of procurement, the ordered medicines may not be available as expressed by one of the mental health provider:

*“Usually, when the medicine reaches at the hospital pharmacy, what we normally do is ordering. When you order the medicine, you find that it is not available. Sometimes when you order, they bring very few medicines for mental health which do not suffice the needs of patients. That’s why you find that we run out of stock* (short of medication) *very often”* (HCW Nurse).

Sometimes the unavailability of medicine was attributed to the small budget set aside for this purpose. This small amount of money could purchase very little medicine needed by a large number of patients who attend the mental health clinic. In addition, the chronic nature of mental illness makes patients use the medications for extended period of time, as explained by a mental health provider:

*“They say perhaps the budget is brought in that way (*inadequate*) but the reality is that psychiatric medication in our country is a problem, it’s not available, and even that little amount brought will not last for long because, I understand psychiatric patients are prescribed monthly dosages. So if 250 cans are bought in a month, will it be enough”?* (HCW SW)

The shortage of psychotropic medication was asserted to be not only a local but a national problem that included other medicines for treatment of various physical illnesses. However, it was mentioned that efforts were being made to make medications available. One of the strategies was to reinstate the essential medicine kits for primary health care and request all hospital to order essential psychotropic medications through cost sharing programs before the government took initiatives to get a permanent solution as stated by national mental health coordinator:.

*“So we are in the process to reinstate the essential medicine kits, at least to reduce the problems of shortage of medicine. In addition, we emphasize that every hospital, even if the issue of budget is small, can order essential psychotropic medicine for people with mental illness through cost sharing when the government is working on how to get sufficient medicines. Hospitals have been given mandate of ordering the medicine that is needed in the departments of mental health according to their needs”* (NMHC).

It was suggested that the Ministry of Health needed to instruct the lower authorities in the health system such as the councils health management team (CHMTs) and regional health management team (RCMTs), so that they set aside enough money in their budget for medicines because they usually set small amounts of money for mental health services. They also suggested that due to the increasing number of patients daily, there should be a basket fund for mental health services that could cover the deficit caused by insufficient funds from the central government.

*“The people in the ministry of health to strengthen the issues of budget in the lower levels so that the district or regional health councils should set aside enough budgets related to mental health” (DMHC)*

Another health care worker suggested:

*“….If we could have our own basket fund for psychiatric patients, we could buy medicine from whole sale traders that could help to get enough medicine for a while” (HCW AMO)*

### Financial concerns towards psychotropic medications

#### Affordability to buy psychotropic medication

Patients, caregivers, health care workers, and coordinators acknowledged the problem of affording to buy medication that was not available in the government pharmacies. The main reason given was poor economic conditions of the patients and caregivers. This was compounded by high prices of medicines in non-governmental pharmacies.

*“I understand that a psychiatric patient is not able to buy* (medicine), *but there are no other ways because that is the reality”* (HCW Nurse)

Some participants expressed that they ought to buy the medication in small quantities depending on the availability of money, otherwise life would be difficult if they would buy all the needed medication dosage at once, as described by of the caregiver:

*“If we buy all the medicine that he uses, life will continues to be difficult because the number of medicine that is required is twenty or thirty, considering that my economic situation is not good. I usually buy them in small amounts like 10 tablets to use”* (FGD 2 CG No 2)

Some patients expressed that they had to miss some doses as a result of inability to buy the prescribed medication especially those from poor families and those without health insurance.

*“…..when you are prescribed a medicine, as you come back home you find that there is no money, and I may stay five, six or seven days without medication until when we get money to buy the medicine”* (Male PT with BPD)

Another patient added:

*“I sometimes get problems because of lack of money, but after several days when we get money, then we buy half a dose”* (Female PT with Schizophrenia)

Although most of the participants described inability to buy prescribed medication due to lack of money, some parents and patients had expressed knowledge of the chronic nature of illness, which they had to set aside a budget that could help the patient when they were required to attend the clinic for follow up care and medicine refill.

*“Well, when I am prescribed with medications, in most cases I usually get prepared for that budget because that is illness, you have to get prepared with money”* (Female PT with Depression)

Health care workers verbalized lack of knowledge about mental illness and the importance of professional treatment by caregivers and relatives as a factor that contributed to unwillingness to provide financial support to patients. Relatives may have the ability to help the patient get the required medication but might be reluctant, as expressed by one health care worker below:

*“They* (the relatives) *do not know the value of mental health. So, if you tell the relative to go and buy medication for the patient who is aggressive, they think, ‘ooh! It is wastage of money’, but he does not know that the drug is beneficial to the patient”* (HCW Nurse)

Some caregivers expressed how they managed to buy medications through assistance from relatives and other people including their partners:

*“Thus, I cannot leave my son alone, I have to do whatever is possible to send him to the hospital, I see different people to beg for financial assistance, they contribute money for sending my son to the hospital” (FGD 2 CG No 7)*

#### Benefits of health insurance

Health insurance was described as beneficial to patients in need of psychotropic medication who could not afford to buy it. The situation for patients who had a health insurance card was much better than those who did not have it. This was said to be more advantageous especially for atypical antipsychotics which are more expensive than the traditional medicines as described by caregivers and a patient:

“…..*when he does not get the medicine there* (hospital pharmacy), *I take his health insurance card and go to take the medicine in a private pharmacy outside the hospital. But for those who depend on out of pocket payment these medicines are very expensive, and patient should not skip a dose, they needs to take every day”* (FGD 1 CG No 5).

*“I do get the medication in the pharmacy using my health insurance card”.* (BPD Female PT)

### Coverage of free treatment policy

The free treatment policy for a mentally ill patient was practiced in the Tanzanian health system. Other groups which benefit from this treatment policy include children, pregnant mothers and the elderly. In this case, psychiatric patients were exempted from all types of costs, including those related to psychotropic medication. They were excluded from cost a sharing system that was used by other groups of patients with physical illness as expressed by one of the health workers below:

*“Our patients for example, they do not pay for psychotropic medicines, treatment or various investigations* etc*. We just fill various needed investigation forms for the patients and send them to the social work office for exemption stamp and then the patients go to collect their medicine in the respective pharmacy. They* (patients) *are excluded from cost sharing system”.* (HCW SW)

Some participants expressed dissatisfaction with the exemption system. It was said to be inadequate due to frequent lack of essential psychotropic medications. Patients and caregivers were expecting free medications that could be available and collected at any time, but this was not possible. Alternatively, they suggested making medications available that could be bought at low price as expressed by a caregiver:

*“It would be better to bring medications that we can buy at low price. I do not know what to do in order to get medications at low price if not for free. We are really getting problems”* (FGD 2 CG No. 9)

Because of the problem of free medication, which was in reality not available, one participant suggested that patients should contribute a small amount of money that could cover the shortage:

*“But if for example, there could be a system for them* (psychiatric patients) *to contribute even a small amount for buying medication at lower price, because here OS* (out of stock) *is too much, we could be able to get stock of medicine”.* (HCW AMO)

At the national level, the coordinator described the strategies that the ministry was implementing to improve the overall health of all Tanzanians. The exemption system was said to have been in place for a long time and was stated in the Tanzanian mental health policy of 2007

*“But the other strategy is the implementation of Mental Health Policy of 2007 that openly proclaims that psychiatric patients should receive treatments free of charge”.* (NMHC)

However, this exemption seemed to be ineffective as the provision of medications was not adequate to cater for the number of patients who were in need. In most cases, the free psychotropic medications were said to be unavailable as described by the district mental health coordinator:

*“Although the mental health services are provided free of charge according to national health policy, getting psychotropic medicine is a big problem especially when you consider that most of patients are very poor”* (DMHC).

## Discussion

This study has illuminated an overview of general challenges related to the availability and affordability of psychotropic medications. Participants who were interviewed acknowledged the therapeutic effects of psychotropic medications when used by the patients and provided several suggestions for meeting the challenges of medication needs.

It is clear that when patients are brought to the health facilities, they have a considerable reduction in symptoms compared to when they are in traditional treatment.

### Positive attitudes towards psychotropic medication

According to the findings from this study, participants appreciated the positive effects of psychotropic medications. This is linked to factors such as perceived efficacy or benefit from medicines and the necessity for taking treatment. It is also due to the fact that most chronic patient with mental illness and their caregivers are adequately educated on the importance of using psychotropic medication. Similar findings have been reported in India [15] indicating that biological model of managing mental disorders outweighs the other treatment modalities and practice. In addition to this findings, De las Cuevas and Sanz in Spain reported that continuous use of psychotropic medication shapes the opinion of the users toward a more beneficial perception of medications [16]. Positive attitudes toward medications among patients and caregivers are the most important aspects likely to augment adherence to medications and prevent relapse of mental illness.

### Availability of psychotropic medication

Accessibility of psychotropic medications is an important facet that needs to be considered in any nation to improve mental health services. According to WHO, access of populations to essential psychotropic medicines is determined by a rational selection of medicines, making prices affordable, ensuring sustainable financing, improved availability and reliable health system and [13].

There are several challenges encountered in meeting metal health needs in most of health facilities in low and middle income countries. The availability of psychotropic medication is one of these challenges as reported in this study. In India, similar findings have been reported indicating that availability, distances and cost of medication were the main challenges that affect utilization of health facilities [14].

This study reported on problems of availability of psychotropic medication, contributed by many factors including inadequate supply. The reasons for inadequate supply of medication reported in this study include delays in the procurement process, priority issues, and inadequate financing. This is similar to the findings reported in Ghana [9], where bureaucracies with the process of procurement also affected the availability and quality of medications. Because of its importance in controlling psychiatric symptoms, patients need to buy the medication outside the hospital pharmacy. The cost of medication when obtained outside the hospital is usually high which has been reported to affect patients socially and financially [17, 18]. Therefore, evidence from this and other studies discussed here, point to the need for strategies to ensure the availability of psychotropic medications in the health facilities. Mental health problems are on the increase, so deliberate actions need to be taken to make sure drugs are available. Evidence in Tanzania revealed that an inadequate supply of medication is one of the factors contributing to relapses of mental illness in most of patients [7]. The results from this study pointed out a need of continuous supply of medication to ensure that patients utilize drugs accordingly. Unavailability of medication in Tanzania is a common problem, also affecting other health sectors including maternal and child health [19]. The unreliability of getting psychotropic medication compromises the timely provision of quality mental health care.

On the other hand, antiepileptic medications were said to be most available compared to other psychotropic medications. The reason for this variation in availability is not known, although in Zambia, social programs aimed at encouraging people with epilepsy to visit health facilities must have increased patient’s access to antiepileptic medication [20]. In Tanzania, patients with epilepsy attend the same outpatient clinic with patients with mental illness and that they all receive the same benefits including free medication as that provided for mental illness. Furthermore, in Gambia and other West African countries, it is reported that lack of demand associated with the considerable cost-implications of treating patient with mental illness and the absence of prescribers are the reason for lack of availability of psychotropic medication [21]. In Tanzania, patients with epilepsy attend the same outpatient clinic with patients with mental illness and they all receive the same benefits including free medication as that provided for mental illness.

Multiple approaches should be used to address challenges within the health system that prevent access to essential medication for mental health. There should be a special focus on improving the governance of the medication delivery system so that it promotes the accountability of key stakeholders, transparency, especially in the dealing with information and medication funds, as well as participation in decision making over the allocation of medication funds.

The government also needs to emphasize on implementation of its policy especially on the use of the basic essential medicine kits that ensure availability of all medicines in primary health services. This could help in alleviating the medicine shortage in most of areas in the country. Although hospitals have been given the mandate of ordering medicines they need, mental health services suffer with lack of financial support. In this study, the hospital management was reported to set aside very low financial budget for mental health. This is partly due to the stigmatizing nature of mental illness among health care providers as reported in other studies [22–24] and partly due to limited budget. There is a need for the ministry of health to increase and strengthen the budget for health in the districts. The council health management team (CHMT) needs to set aside enough money in the budget for mental health to help in improve the availability and accessibility to psychotropic medications.

### Financial concerns towards psychotropic medications

Health insurance for mental health in many low- and middle-income countries has not yet been extensively practiced. The goals of health insurance are to make health services more affordable through the use of public subsidies. In this study, most mentally ill patients were not covered by national health insurance scheme. This is because the coverage of national health insurance scheme is very limited. According to the Ministry of Health, Community Development, Gender, Elderly and Children (MoHCDGEC), the l health insurance scheme for public servants covers only 20% of the total population. Medicines are covered by insurance but its availability in the treatment facilities is a which needs to be addressed [25]. In other countries, studies have shown strong evidence that health insurance increases financial protection by reducing out-of-pocket spending [26] which relieves patients with mental illness

It was reported in this study that patients and caregivers could not afford to buy medication due to the poor economic situation of the family members and the patient. This can be explained by the fact that the mental disorders usually are chronic diseases that make the patients and caregivers spend too much of their savings to treat the illness. Cost analysis in India revealed that some specific brands of psychotropic medication are expensive and difficult to buy even though it is effective for alleviating the symptoms of mental illness [18]. This reveals an important aspect which is for prescribers to be aware of the ability of their patients and caregivers to afford that medication before prescribing.

It is also reported that strong social and financial support increases the likelihood of the patient buying medications. The support from relatives or other people around the patient is needed, as it is important both for physical and psychological relief, especially when directed towards assistance in buying prescribed medication. It is known that lack of support for patients with mental illness living in the community is a problem [27, 28]. The reason for this in Tanzania is not fully understood. Most of patients and caregivers in this study have reported poverty as contributing factor. In Ghana, the caregivers indicated that lack of surplus money, stigma, and lack of empathy to be important factors contributing to poor social and financial support [29].

It is difficult to provide financial support to the patients, especially that which is needed for medication. Even when the support is available, it cannot be sustained because mental illness tends to be chronic and needs a continued supply and use of prescribed medication.

### Coverage of free treatment policy

It revealed from this study that people with mental illness are included in free treatment package according to the National Health Policy [30]. The other group includes the elderly, children and maternal health services. According to this policy, these are the low income population who obtain their subsistence through their dependants. The patients in these groups are exempted from cost sharing and other related health charges such as lab investigations and consultation fees. However, the coverage of this service is very minimal with respect to patients in need. The reasons for ineffective exemption policy in Tanzania is not clearly known although lack of commitment of health managers, confusion about the eligibility criteria, failure of central government to establish clear eligibility criteria and limited technical support are some of the reasons reported in other studies [31, 32]. The availability of free treatment has not been realistic in terms of access to free medications for patient with mental illness. Inadequate supply of medication is the main stumping stone for quality mental health services.

### Limitation of the study

The lack of psychiatrists in the participant group is one of the limitations of the study. This would probably give different perspectives and expertise on areas of prescribing practice. The study is also limited by lack of pharmacists in the participant group who could discuss the experience of dispensing psychotropic medication. Finally, the study is based on only one district which may have limited representation of the other districts in the country.

## Conclusion

Availability and affordability of psychotropic medication for patients were shown to be inadequate in this study. This was attributed to insufficient funds to support the health facilities and technical challenges contributed by both by the health facilities and stakeholders. To improve mental health services in the country, it is important to ensure adequate and regular supply of psychotropic medications for mental health. Essential psychotropic medicines were usually unavailable in the health facility. Access to psychotropic medications is essential to address the public health burden attributed to untreated mental illnesses. These findings call for the government to increase funds allocated specifically for buying essential psychotropic medications and improving the process of procurement and supply.

## Abbreviations

LMICs: low and middle income countries
WHO: World Health Organization
FGDs: focus group discussions
MHCW: mental health care workers
CG: caregivers
PT: patients
AMO: assistant medical officers
DMHC: district mental health coordinator
NMHC: National mental health coordinator
